# Consensus in Action: Context-Specific Physical Activity Guidelines for Undergraduate Students at a South African University

**DOI:** 10.3390/ijerph21121651

**Published:** 2024-12-10

**Authors:** Chanté Johannes, Nicolette V. Roman, Sunday O. Onagbiye, Simone Titus, Lloyd L. Leach

**Affiliations:** 1 Department of Sports, Recreation, and Exercise Science, University of the Western Cape, Cape Town 7535, South Africa; sonagbiye@frederick.edu (S.O.O.); titusdawsons@sun.ac.za (S.T.); lleach@uwc.ac.za (L.L.L.); 2 Centre for Interdisciplinary Studies of Children, Families and Society, University of the Western Cape, Cape Town 7535, South Africa; nroman@uwc.ac.za; 3 Department of Health and Exercise Sciences, Frederick Community College, Frederick, MD 21701, USA; 4 Department for Health Professions Education, Faculty of Medicine and Health Sciences, Stellenbosch University, Cape Town 7505, South Africa

**Keywords:** physical activity, guidelines, consensus workshop, context-specific, undergraduate, university students, South Africa

## Abstract

Physical inactivity among undergraduate university students has been considered a public health concern. To address this, researchers have utilized consensus workshop approaches to develop effective physical activity (PA) recommendations. However, the existing research has limitations: it is outdated, not context-specific to young adults, and does not account for psychosocial factors (such as mental health, motivation, and social support) that hinder or promote PA behavior, particularly in South Africa. Therefore, the purpose of this study was to engage with stakeholders to achieve a consensus on a set of context-specific guidelines to enhance the physical activities of undergraduate university students. Utilizing the Social Ecological Model, this study employed two online consensus workshops with 25 purposively selected stakeholders (Round 1 = 8 and Round 2 = 17). Stakeholders were divided into breakout rooms via the Google Meets feature, to discuss and brainstorm the guidelines, expressing their agreement or disagreement with the proposed names and descriptions. The consensus was considered achieved when the majority of stakeholder responses fell into the ‘Agree with the guideline’ category. An inductive thematic analysis approach was used to generate common themes, which were then coded via Atlas Ti. V8. Stakeholders reached a consensus on four categories and 32 guidelines, namely, PA (9 guidelines), mental health (7 guidelines), motivation (9 guidelines), and social support (7 guidelines). Each category, along with its respective set of guidelines, provides insights into the type of information undergraduate students require to enhance their PA participation. Using a consensus workshop facilitated the co-creation of context-specific guidelines to enhance the physical activities of undergraduate university students. This approach proved to be a valuable tool for fostering collaboration between academic staff and students.

## 1. Introduction

Physical inactivity and sedentary behavior have been reported as the fourth leading risk factor that contributes to global mortality [1,2]. According to Pogrmilovic et al. (2020), over the years, national and subnational governments, international organizations such as the World Health Organization (WHO), public health researchers, and non-governmental organizations have undertaken various initiatives to promote physical activity (PA) and reduce sedentary behavior, recognizing it as a public health priority [3]. Despite vast research on the benefits of PA, detrimental healthcare challenges continue to rise among physically inactive populations [4,5]. For example, the overall PA status among young adults, such as undergraduate university students, is insufficient to maintain an active lifestyle [6] and although university students understand the benefits of PA, their positive attitudes do not necessarily translate into changing their sedentary behavior [7]. Sedentary behavior, according to research, is said to be exceptionally high among university students [8]. In addressing sedentary behavior, stakeholders and policymakers have utilized the consensus workshop approach to developing effective PA strategies and guidelines. Previous studies have utilized the consensus workshop approach to address topics related to PA among children and adolescents [9], measures to assess mental health and sport [10], and PA questionnaire validation [11] and to explore the role of family and PA and sedentary and sleep behavior [12]. Additional researchers have explained the conceptualization and implementation of consensus workshops and their value for collaboration and co-creation between stakeholders [13,14]. Nevertheless, this workshop approach has rarely been employed or has been regarded as outdated within the South African context [15].

In terms of South Africa (SA), the last consensus workshop approach utilized was conducted in 2012, centering on the Vuka SA initiative aimed at promoting PA across the nation [15]. This research paper delved into the initiative, highlighting the absence of a comprehensive national plan for PA promotion prior to Vuka. Previously, programs operated independently with limited evaluations. The Vuka initiative sought to expand the reach of PA messaging, implement supportive policies, shift social norms toward PA, and increase participation rates. Inspired by successful global campaigns, Vuka SA aimed to tackle the escalating global concern of non-communicable diseases associated with physical inactivity [15]. However, the limitations of this research are that it is outdated, not context-specific to young adults, and does not include psychosocial factors that hinder or promote PA behavior. This highlights the need for updated context-specific guidelines that address the unique challenges and needs of young adults, particularly undergraduate university students.

While previous studies have utilized consensus workshops to develop PA recommendations and policies, there is a noticeable research gap. Limited studies have employed this approach to develop guidelines to enhance the physical activities of undergraduate university students in SA, where physical inactivity remains a challenge [16]. Previous studies have either (1) primarily focused on children and adolescents [9], (2) centered around mental health [10], (3) focused on physical education within the school curriculum or classroom setting [16], or (4) involved generic PA guidelines and recommendations that could be used across various age groups, ranging from children to older adults [15]. Hence, there is an opportunity to utilize a consensus workshop approach to develop context-specific PA guidelines for undergraduate university students. The research gap addressed by this study lies in the lack of evidence-based PA guidelines for undergraduate university students at a South African university. While much of the existing literature focuses on general PA recommendations [15], it often overlooks the unique challenges students encounter (such as time constraints and lack of knowledge) [7] and psychosocial factors that influence student engagement (such as mental health, motivation, and social support). By incorporating psychosocial factors into the context-specific guidelines, this research provides a novel contribution to the literature. Therefore, this article describes the process of the consensus workshop that involved the co-creation and collaborative effort of stakeholders (i.e., undergraduate university students and experts in the field of PA, sports science, and public health) to achieve consensus on the development of context-specific guidelines to enhance the physical activities of undergraduate university students.

### Development of the Guidelines and Conceptual Framework

**Figure 1:**
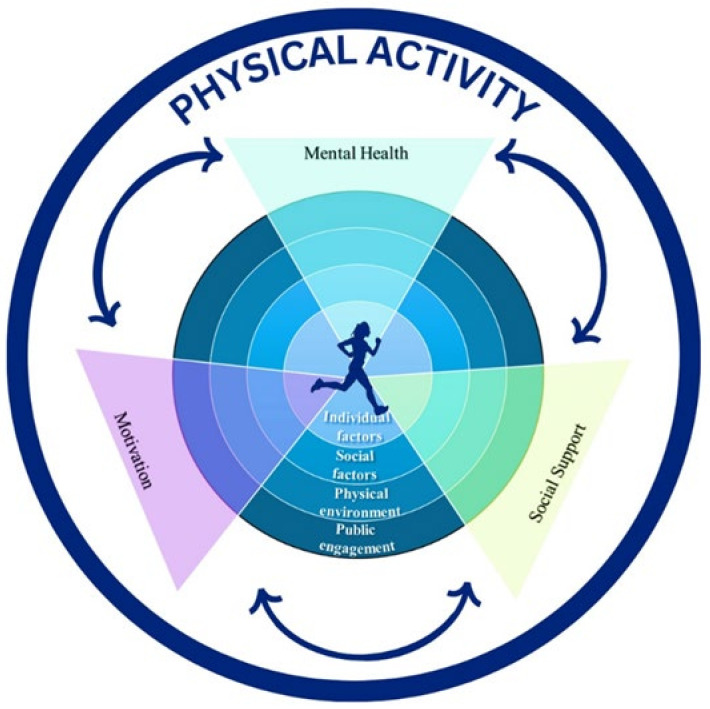
Adapted Social Ecological Model, which consists of four levels: individual factors, social factors, the physical environment, and public engagement. Physical activity (PA) is the central theme, represented by the encompassing circle, and is influenced by human behavior, shaped by psychosocial factors such as mental health, motivation, and social support. These interconnected psychosocial factors collectively influence a student’s behavior and decision to engage in PA.

Before the consensus workshop began, a mixed-methodological approach employing a sequential explanatory research design was utilized. This approach aimed to establish the foundation for conceptualizing the consensus workshop and to identify the PA levels, preferences, and psychosocial factors among undergraduate university students. The current study was part of a broader study in which the stages and phases included participants as follows. In Stage 1 a total of *n* = 534 undergraduate students completed the quantitative questionnaire. In Stage 2, *n* = 18 Students participated in the qualitative interview process. The findings derived from the mixed-methodological approach informed the development of the PA guidelines for the consensus workshop. In order for the PA guidelines to be context-specific, the Social Ecological Model (SEM) was employed. The SEM serves as a comprehensive framework for understanding and addressing health behaviors such as PA within various contexts [17]. This model recognizes that individual behavior is influenced by multiple levels of influence, ranging from individual factors, social factors, and the physical environment to public policy [18,19]. Based on the findings derived from the mixed-methodology studies and in the context of developing PA guidelines for the consensus workshop, the SEM was adapted (to individual factors, social factors, the physical environment, and public engagement), which guided the integration of findings from the mixed-methodological approach (Figure 1). Based on the findings derived from the mixed-methodological research, which identified social media and public engagement as key factors, the term public engagement was deemed more appropriate than public policy within the context of the SEM. In adapting the SEM for this current study, focus was placed on incorporating elements of public engagement to reflect the role of digital platforms and public participation. This adaptation was made to ensure the SEM captured the dynamic context of social media’s influence on public discourse.

Considering these multiple levels of the SEM, the guidelines were tailored to address psychosocial factors such as mental health, motivation, and social support. These interconnected psychosocial factors collectively influence a student’s behavior and decision to engage in PA [20]. This approach ensured that the guidelines were context-specific, considering the unique challenges and opportunities present within the university setting [21]. From the results obtained from the mixed-methodological approach, the guidelines were split into four categories, namely PA (consisting of 10 guidelines), mental health (consisting of 8 guidelines), motivation (consisting of 10 guidelines), and social support (consisting of 7 guidelines). Thus, a cumulative total of 35 guidelines were generated. Table 1 shows the findings from the mixed-methodological approach, utilizing the sequential explanatory research design, which was captured and reorganized into guidelines within the adapted version of the SEM framework. Thus, employing the SEM facilitated a holistic understanding of PA promotion, which directly supported the study’s objectives of engaging with stakeholders to reach a consensus on a set of context-specific guidelines, aimed at enhancing the physical activities of undergraduate university students. This approach ensured the relevance and applicability of the guidelines for improving student health and well-being.

## 2. Materials and Methods

The consensus workshop aimed to develop context-specific guidelines to enhance the physical activities of undergraduate university students. The objective of this study was to engage with various stakeholders (i.e., undergraduate university students and experts in the field of PA, public health, and exercise) to reach a consensus regarding the topics that emerged from the mixed-methodological process.

### 2.1. Ethics Considerations

Ethics approval and permission from the university were obtained prior to the commencement of this study, reference number: HS21/10/24. All stakeholders who voluntarily participated in the consensus workshop provided their consent and signed a confidentiality binding form. Before the start of the consensus workshop, stakeholders were reminded that their participation in the study was voluntary and that they may stop and withdraw at any stage during the workshop, without any penalty. To ensure confidentiality and anonymity, participants’ names were removed and replaced with pseudonyms.

### 2.2. Study Design

The consensus workshop employed a qualitative research approach, which has previously been recognized as a valuable method for conducting qualitative research [14]. A consensus workshop is an innovative participatory group approach where group discussions are facilitated, ensuring that all participants have an opportunity to contribute and influence the outcome of a single jointly held discussion. Thereafter, the discussion of the workshop is analyzed and reported [16]. This approach was selected for this study to achieve consensus, assess weaknesses and alterations, and determine whether the PA guidelines are context-specific and valid and if they address the main aim of this study. This technique is advantageous because it is an anonymous process, as stakeholders are able to contribute their judgments and opinions in a free and open manner [22].

### 2.3. Study Setting and Participants

Stakeholders for this study were drawn exclusively from a single university located in the Western Cape Province of SA. The choice of a single university setting enabled a detailed exploration of contextual factors directly pertinent to the research objectives. Universities operate within diverse psychosocial contexts, and focusing on a specific institution facilitated a deeper investigation into the unique psychosocial dynamics present within the university environment. This approach allowed for a comprehensive examination of the PA among undergraduate students, enhancing the study’s relevance and contextual richness. Stakeholders in the field of PA were purposively selected to participate in the consensus workshop. Within a consensus workshop, a maximum of 40 stakeholders may be considered [16]; however, the larger the sample, the lower the feedback and consensus rate [22]. The selection criteria employ the purposive sampling technique as the stakeholders (i.e., undergraduate university students and experts in the field of health) were purposefully selected based on the inclusion and exclusion criteria. Regarding the inclusion and exclusion criteria, the stakeholders included in this study were undergraduate university students across different academic programs, degrees, and faculties as well as academic doctors and professors. For undergraduate students, the inclusion criteria encompassed all genders, students registered at a university in the Western Cape Province of SA, and those aged 18 years and older. Additionally, students who had participated in the mixed-methodological studies and provided consent were included. The exclusion criteria comprised students registered for non-degree purposes or only for one semester, those under the age of 18, and those who had not participated prior in the mixed-methodological studies or did not provide consent. Academic doctors and professors included in the study were those based in SA, specializing in PA, sports science, or public health, with a minimum of 20 peer-reviewed publications in these fields. Professors or doctors not specializing in these areas, with fewer than 20 publications, or based outside SA, were excluded. All stakeholders were required to provide consent for participation.

The consensus workshop occurred in two rounds. In Round 1, the study facilitator extended invitations via email to 12 potential academic stakeholders from a University in the Western Cape province of SA (see Table 2 for the inclusion criteria). The selection of 12 academic stakeholders allowed for the possible withdrawal of some, since all academic stakeholders possessed similar professional experiences as mentioned in the inclusion criteria. Two invitations were sent via email to stakeholders to participate in this round. A total of eight stakeholders participated in Round 1. This smaller group size facilitated in-depth discussions from diverse perspectives. In Round 2, the facilitator invited 30 academic doctors and professors and 22 undergraduate university students (Table 2), to broaden the scope of input and to enhance the robustness of the findings while keeping the group size manageable for meaningful participation and consensus-building. Five invitations and reminder emails were sent to stakeholders to participate in this consensus round. A total of 17 stakeholders voluntarily consented to participate in this study in Round 2. Therefore, a cumulative total of 25 stakeholders participated in this study.

### 2.4. Data Collection Process

**Figure 2:**
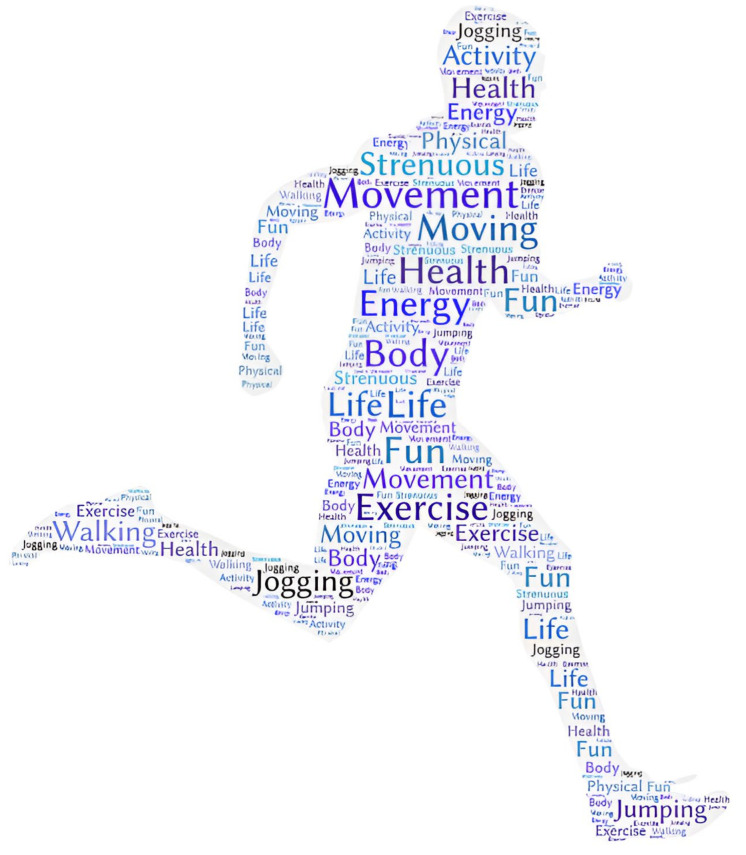
Keywords as described by stakeholders in the consensus workshops.

The consensus workshop was conducted online in English (as this is the language of instruction at the institution) with the stakeholders. Two rounds of consensus workshops were hosted. Round 1 occurred in July 2024 and Round 2 commenced in August 2024. Both workshops lasted approximately 3.5 h long. Workshops were conducted via the Google Meets digital platform (as part of the coronavirus precautionary measures). With the participants’ consent, the workshops were audio-recorded. In the structured format of a consensus workshop [16], the primary researcher assumed the role of facilitator, guiding the process through defined rounds. Initially, the facilitator set the context by introducing and outlining the workshop’s aim, objectives, and procedural framework. Additionally, stakeholders were informed that consensus on the proposed guidelines would be reached when a minimum of 75 % of responses fell into the category ‘Agree with the guideline’ [16,22]. Stakeholder engagement began with an icebreaker where participants had to describe PA in one word. In a consensus workshop, starting with an icebreaker allows participant interaction, creates a comfortable environment for open dialogue and collaboration, and assists the facilitator in understanding the collective understanding of PA as described by the participants [23]. Figure 2 (created using WordArt.com, 2009, https://wordart.com/ (accessed on 20 November 2024)) shows the keywords that stakeholders utilized to describe PA.

**Figure 3:**
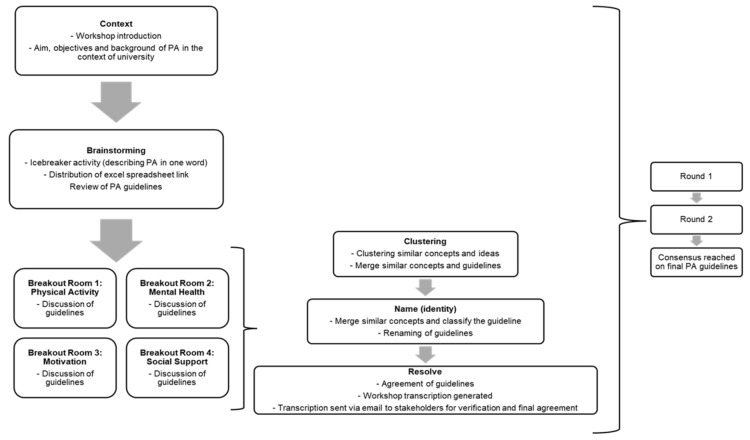
Consensus workshop process.

Next, the facilitator provided stakeholders with a link to the Google Excel spreadsheet where the guidelines were stored, enabling stakeholders to access and review them as they deliberated within their respective breakout rooms. Stakeholders were divided into breakout rooms to discuss and brainstorm the guidelines, expressing their agreement or disagreement with the proposed names and descriptions. The breakout rooms consisted of approximately 4–5 stakeholders in each room, focusing on specific themes: Room 1 addressed PA, Room 2 focused on mental health, Room 3 explored motivation, and Room 4 discussed social support. A technical assistant was present throughout the meeting to facilitate smooth proceedings of the breakout rooms. Moving to the identity formation stage, the facilitator guided stakeholders in generating options and suggestions. As discussions progressed, similar topics were merged, and consensus-building discussions were facilitated to resolve any discrepancies. Finally, the facilitator oversaw the voting process (stakeholders had to indicate ‘*Agree with the guideline*’ or ‘*Disagree with the guideline*’ on the Excel spreadsheet for their vote to be tallied). This process was repeated for both rounds of the consensus workshops. In Round 2, the consensus was considered achieved when a minimum of 75% of responses fell into the category ‘*Agree with the guideline*’ [24]. Once the consensus workshop process was completed, the session was transcribed by the primary researcher and emailed to the respective stakeholders to provide them with an opportunity to review the information presented and to supplement any additional details that might have been overlooked during the workshop. Three email reminders across 15 days were sent to participants to verify the transcript’s accuracy and confirm the information provided. This resulted in the development of final written guidelines based on the consensus reached during the workshop. Thereafter, the data analysis phase commenced. Figure 3 shows the steps in the consensus workshop process.

### 2.5. Data Analysis

For this qualitative study, inductive thematic analysis was employed. Thematic analysis helps researchers to identify, analyze, and group data that have similar patterns (themes) [25]. This approach was appropriate as it allowed for important information to be generated to reach the objectives of this current study. Recordings from the interviews were transcribed verbatim by the first author and uploaded to a qualitative analysis software, namely Atlas Ti. V8. Qualitative themes were developed based on the following four phases: initialization, construction, rectification, and finalization [26,27]. The initialization phase involved reading and re-reading transcriptions, highlighting significant meanings, coding, and seeking abstractions in participants’ accounts, along with writing reflective notes. The construction phase entailed classifying and comparing the data, labeling the findings to provide context, and defining and describing the emerging codes and themes. Throughout the rectification phase, the researcher practiced immersion and distancing (also known as bracketing) to self-correct and align the study findings with established knowledge, formulating thematic statements linked to previous research. Lastly, the finalization phase focused on developing a narrative that tied the themes to the existing literature, thus enriching the understanding of the phenomenon in the context of this study. Once the themes were created, a thematic table was developed. This table was modified numerous times where themes and subthemes were combined based on similarity. For example, key findings from the mixed-methodological study found that 29% of undergraduate students were physically inactive, 31.1% were minimally active, and 39.9% were in the health-enhancing category [28]. Other notable results included a preference for evening sessions and endurance activities [28]. These results were then translated into common themes, such as the need for gradual progression, the importance of sustaining and diversifying activities, and a preference for activities that align with students’ schedules and interests. For instance, the finding that a significant portion of students were physically inactive or minimally active led to the development of Guideline 1: Start with small beginnings and Guideline 2: Increase activity levels gradually. The preference for endurance activities informed Guideline 6: Choose preferred activities, while the preference for evening sessions resulted in Guideline 5: Schedule PA strategically. This process ensured that the guidelines were directly informed by the mixed-methodological findings, translating the themes into practical actionable recommendations for enhancing PA among undergraduate students. Thereafter, the thematic table was finalized and all authors agreed on the final set of themes that were relevant to the study’s objectives [28].

### 2.6. Trustworthiness

Trustworthiness can be measured through four criteria: credibility, transferability, dependability, and confirmability [29,30]. For credibility, triangulation is utilized in order for the research findings to be considered credible. This study’s credibility was achieved using triangulation (various sources and methods to corroborate findings) and member checking (where feedback was obtained from the workshop participants to validate the transcriptions). The descriptions, interpretations, and results were made available to participants to determine the information’s accuracy. Workshop participants were provided two weeks in which to respond with corrections and feedback [31]. Transferability entails providing readers with evidence that the study’s findings could apply to additional contexts [28,32]. For this study, transferability was achieved by employing a thick description, where the researchers ensured that sufficient information was presented for the results to be relevant and applicable to similar contexts [33]. Dependability ensures that the study’s results remain consistent and dependable over time and under different conditions [34]. This study ensured dependability by grounding the interpretation in the data analysis itself, rather than relying on the researcher’s perspective. Confirmability suggests that the findings are objective and not influenced by the researchers’ preconceived ideas and biases [29]. For this study, confirmability was achieved as researchers were transparent about their perspectives and biases and kept an audit trail (where decisions were documented to allow for scrutiny and verification) [32]. The audit trail was maintained by documenting all methodological decisions, data collection processes, and thematic developments in a research journal. It also included reflections on analytical choices and justifications for any adaptations made during the study. This trail was actively utilized throughout the research process to ensure the consistent application of methods, track the progression of analysis, and maintain alignment with the study’s objectives. By providing a clear and thorough account of the research process, the audit trail enhanced transparency and minimized the influence of researcher subjectivity, thereby strengthening the overall trustworthiness of the research process [32].

## 3. Results

### 3.1. Sociodemographic Details

Table 2 shows the sociodemographic information of the stakeholders who participated in the consensus workshops. In Round 1, a cohort of eight staff members was involved with the majority being female (*n* = 5, 62.5%). The participants included a social work supervisor, information manager, academic manager, professor, associate professor, senior lecturer, lecturer, and senior sports organizer. Their respective fields of expertise were diverse, encompassing social work, health informatics education, student-athlete support and sports psychology, child and family studies, health professions education, medical biosciences, and sport management and excellence. In Round 2, eight students participated in the workshop, with the majority being female (*n* = 5, 62.5%), from the Community and Health Sciences faculty (*n* = 6, 75%) and in their third year of study (*n* = 5, 62.5%). The student cohort included individuals specializing in commerce, law, sports, recreation and exercise sciences, social work, and physiotherapy. Additionally, the second round involved nine staff members, with the majority being female (*n* = 6, 66.7%). This group consisted of lecturers, an associate professor, a head of a department, senior lecturers, and a director. These participants brought expertise in fields including physiotherapy, sports sciences, sports management, biokinetics, recreation, leisure and disability, high-performance sport, exercise physiology, sports sociology, and mental and public health.

**Table 2:** Sociodemographic information of the stakeholders who participated in the consensus workshops.

ROUND 1					
**Academics (*n* = 8)**					
**Classification**	Sex	Ethnicity	Position	Field of Expertise	Department
**Staff**	Female	Coloured	Social work supervisor	Social work	Social Work
	Male	African	Information manager	Health Informatics Education	Information management
	Male	White	Academic manager	Student-athlete support and sport psychology	Non-government organisation within the university
	Female	Coloured	Professor	Child and Family Studies	Child and Family
	Female	Coloured	Associate Professor	Health Professions Education	Health Professions
	Female	Coloured	Senior Lecturer	Social Work	Social Work
	Male	Indian	Lecturer	Medical Biosciences	Medical Biosciences
	Female	African	Senior Sports Organiser	Sport Management and Excellence	Sports
**ROUND 2**					
**Undergraduate University Students (*n* = 8)**					
**Classification**	Sex	Ethnicity	Faculty	Department	Year
**Student**	Female	African	Economic and Management Sciences	Commerce	3rd year
	Male	African	Law	Bachelor of Law	4th year
	Male	Coloured	Community and Health Sciences	Sports, Recreation and Exercise Science	3rd year
	Female	Coloured	Community and Health Sciences	Sports, Recreation and Exercise Science	3rd year
	Female	African	Community and Health Sciences	Social Work	4th year
	Female	African	Community and Health Sciences	Physiotherapy	3rd year
	Female	African	Community and Health Sciences	Social Work	4th year
	Male	Coloured	Community and Health Sciences	Sports, Recreation and Exercise Science	3rd year
**Academic Doctors and Professors (*n* = 9)**					
**Classification**	Sex	Ethnicity	Position	Field of Expertise	Department
**Staff**	Female	Coloured	Lecturer	Physiotherapy	Physiotherapy
	Female	White	Associate Professor	Sports Sciences	Sports, Recreation and Exercise Science
	Male	Coloured	Lecturer	Sports Management, Sport Sciences, and Biokinetics	Sport Management
	Female	White	Lecturer	Sport Management and Sport Sciences	Sport Management
	Male	African	Lecturer	Recreation, Leisure, and Disability	Sports, Recreation and Exercise Science
	Male	White	Head of Department and Senior Lecturer	Sports Sciences and High-Performance Sport	Sports, Recreation and Exercise Science
	Female	Coloured	Lecturer	Exercise Physiology and Sport Sociology	Sport Management
	Female	Coloured	Director	Mental Health	Therapeutic Services
	Female	Coloured	Senior Lecturer	Public Health	Public Health

Note: The term ‘Coloured’ in the South African context describes individuals with mixed ancestry.

### 3.2. Consensus Workshop—Round 1

Table 3 shows the first round of recommended guidelines with comments from the stakeholders. This table indicates the original set of guidelines that were developed by the primary researcher. The goal of Round 1 was to share the following with the stakeholders: (1) to describe the aim and objectives of the consensus workshop, (2) to present the findings derived from the sequential explanatory mixed-methodology studies and lastly, and (3) to ascertain whether the guidelines resonate with the findings of the mixed-methodological results. Stakeholders were asked to respond to a set of questions and statements, namely, (1) indicate which guidelines were suitable and resonated with the mixed-methodological research findings, (2) comment on the language used to describe the guidelines, for it to be easily understood by undergraduate university students, and (3) establish if any additional information that needed to be included in the guidelines are required. In response to the above set of questions, the following consensus was reached by the stakeholders:

1. As a result of similarities found within some of the findings, the stakeholders agreed to the merging of several guidelines within the PA category, specifically Guideline 10 had to be merged with Guideline 4. This resulted in 9 guidelines within the PA category;
2. The stakeholders agreed that some guidelines (for example, guidelines 2 and 4 within the mental health category) should be reworded to more simplistic terms;
3. Stakeholders agreed with the guidelines and no additional guidelines were proposed.

Stakeholders unanimously agreed on proposed categories and their respective guidelines. A final consensus was reached on four categories with 34 overall guidelines. Once Round 1 was completed and stakeholders provided their final input, the guidelines were revised according to the recommendations and were prepared for Round 2.

### 3.3. Consensus Workshop—Round 2

Table 4 shows the second round of the consensus workshops with stakeholder comments and suggestions. This table shows the guidelines that were revised by the primary researcher. The goal of Round 2 was to generate further feedback on the PA guidelines derived from Round 1. Additional objectives were (1) to share the aim and objectives of the consensus workshop with the stakeholders, (2) based on the revisions made in Round 1, to identify additional guidelines that resonate with the findings obtained from the mixed-methodological research. The stakeholders were asked to respond to a set of questions corresponding to revised PA guidelines, namely, to (1) indicate if the guidelines were suitable for undergraduate university students, (2) indicate if the language used to describe the guidelines could be easily understood by undergraduate university students and lastly, and (3) establish if there was there any additional information that needed to be included in the guidelines. In response to the above set of questions, the following consensus was reached by the stakeholders:

1. Based on the recommendations made in Round 1 and Round 2, the stakeholders agreed that the guidelines were suitable for undergraduate university students;
2. The stakeholders agreed that some guidelines (for example Guidelines 1 and 3 from the motivation category) should be reworded to more simplistic terms. Additionally, guidelines 4 and 5 within the motivation category should be combined due to the similarity in concepts;
3. Stakeholders agreed with the guidelines and no additional guidelines were proposed.

Once Round 2 was completed and stakeholders provided their input, the guidelines were revised according to the recommendations. Thereafter, the stakeholders reached unanimity on the four categories (PA, mental health, motivation and social support) with 32 guidelines overall.

## 4. Discussion

The purpose of this study was to engage with stakeholders to achieve consensus on a set of context-specific guidelines to enhance the physical activities of undergraduate university students. The guidelines were co-created with the input of undergraduate students and academic experts, who ensured they were context-specific and relevant to students from a South African university. The guidelines developed in this study highlight a holistic approach to enhancing PA among students. Four categories were agreed upon, namely, PA, mental health, motivation, and social support. Each category comprised a distinct set of guidelines. For instance, the PA category consisted of nine guidelines: mental health included seven guidelines, motivation included nine guidelines, and social support encompassed seven guidelines. Overall, consensus was achieved across the four categories, with 32 guidelines. Additionally, stakeholders provided suggestions regarding the language and tone of the guidelines.

The sociodemographic characteristics of the stakeholders, including gender, ethnicity, and academic discipline, played a significant role in shaping the final guidelines. The diverse academic backgrounds of the participants—spanning disciplines such as physiotherapy, sports sciences, sport management, and public health—provided a well-rounded perspective on the various facets of PA and its relevance to the student population. Insights from stakeholders with expertise in exercise physiology, mental health, and sports management highlighted the need for clear accessible language to ensure the guidelines were inclusive and applicable to students with varying levels of knowledge of PA. Furthermore, the gender and ethnic diversity among participants contributed to the development of culturally relevant and gender-sensitive recommendations [35,36]. This sociodemographic diversity directly influenced the terminology, tone, and content of the guidelines, ensuring that they were practical, easy to understand, and tailored to undergraduate students. Nevertheless, it should be noted that since the focus of the study was on PA, the participants from sport-related programs were predominant, as these students are enrolled in disciplines where PA is central to their curriculum. Students in these programs are often more engaged with PA and possess a greater understanding of its terminology, making them more likely to participate in the study. However, this concentration may limit the diversity of perspectives. Future research could benefit from including participants from a wider range of academic programs to ensure the broader applicability of the findings. This would also help balance the language and tone of the guidelines, which may have been influenced by the participants’ specific backgrounds in sports.

Stakeholders suggested that the guidelines be consistent in terms of language use. For example, they recommended standardizing terminology across all guidelines to avoid confusion, such as consistently using the term ‘PA’ instead of alternating with ‘exercise’ (as ‘exercise’ may be interpreted as a vigorous sport and thus some students may not want to participate in sport, but rather engage in healthy activities such as stretching). Additionally, stakeholders suggested that the guidelines should be kept ‘simple’. For instance, the guidelines were developed for undergraduate university students; thus, complex terminology such as ‘master’ (in the motivation category) would be difficult to understand. Therefore, the term ‘set’ was recommended as an alternative. This ensured that the guidelines were easily understood, which were agreed upon by the students in Round 2. To complement the language and tone of the guidelines, stakeholder comments were further scrutinized to enhance the guidelines within their respective categories.

In terms of the PA category, stakeholders from both rounds reached a consensus on nine guidelines. These guidelines focused on small beginnings, gradually increasing activity levels, and sustaining and diversifying activities, emphasizing the importance of a progressive and sustainable approach to PA. From the stakeholder comments, two key aspects were emphasized. Firstly, an overarching note should stipulate that the modalities as recommended in the guidelines are purely PA-based and have no link to religion or spiritual background. Secondly, another note should emphasize that people with mobility impairments may explore alternative forms of PA that are safe. These suggestions corroborate the findings of previous research where it was indicated that incorporating aspects of disability, spirituality, and religion is crucial for promoting inclusive and effective health behaviors and interventions among diverse populations [35,36]. By integrating these inclusive elements into the guidelines, the study builds on established research to ensure that diverse needs are met. Therefore, the guidelines presented in this study have the potential to enhance PA levels by promoting education, diversifying activities, and encouraging monitoring and tracking [37].

Regarding the mental health category, undergraduate university students and academic experts reached a consensus on seven guidelines. Guidelines focused on mental health, such as using PA as a coping mechanism for depression, reducing anxiety, and relieving stress. This highlights the important role of PA in managing mental health [38]. Notably, based on the stakeholder comments, three main aspects were suggested. Firstly, to begin a PA journey, individuals need to foster a motivational mindset of ‘getting in the zone’. This suggestion was similar to another study where it was indicated that a positive mindset is associated with increased PA levels as it promotes resilience and empowers individuals to navigate challenges effectively [39]. Secondly, social interaction among peers was considered a crucial factor, as more discussions are needed concerning mental health. Similarly to previous research, peer engagement has been shown to significantly enhance students’ sense of belonging, which is crucial for their overall well-being and mental health, particularly for those who may feel isolated in the university environment [40]. This suggests that mental health should be destigmatized and incorporated into everyday conversations among students [41]. Lastly, mental health illnesses such as depression, anxiety, and stress should be described in layman’s terms so that they can be easily understood by undergraduate students. The need for layman’s terms concurs with previous studies, which recommended that describing mental health illnesses in layman’s terms is crucial for enhancing public understanding and reducing stigma [42]. For instance, many individuals associate mental disorders with emotional distress and impairment but may not recognize common conditions such as anxiety. This disconnect could hinder help-seeking behaviors [43]. Thus, it is envisioned that the guidelines derived in this study will not only promote PA as a coping mechanism but also serve as a foundation for future research to explore their long-term influence on student well-being and the effectiveness of their implementation across diverse university contexts.

In terms of the motivation category, stakeholders from each round of the workshop agreed on nine guidelines. This category addressed the need for both intrinsic and extrinsic motivational factors, setting motivational goals, and incorporating enjoyable activities. Additionally, the inclusion of social media as a tool for fostering a healthy mindset and engaging in positive fitness challenges reflects the reality of students’ digital lives and offers a way to harness technology for health promotion [44]. Specifically, according to stakeholder comments, the guidelines should consider being inclusive of students at various stages of their fitness journey. These comments are consistent with previous studies where it was indicated that inclusivity promotes equity and thus ensures that every student has the opportunity to participate in PA and benefit from the positive health effects [45,46]. It is envisioned that by recognizing and accommodating these differences, the guidelines could support all students in achieving their personal health goals, fostering a more supportive and effective environment for overall well-being [47]. Thus, inclusive guidelines are crucial for fostering a more active and healthier society. Building on this foundation, future research could focus on evaluating the implementation of these inclusive guidelines, particularly their effectiveness in promoting equitable access to PA and their broader impact on undergraduate university students’ well-being.

Regarding the social support category, seven guidelines were agreed upon in both consensus workshops. The social support guidelines emphasize the importance of community and peer networks in promoting PA, surrounding oneself with support networks, promoting community wellness, and utilizing campus recreational resources. From the stakeholder’s perspectives, it was reiterated that the terminology ‘family’ should change to ‘community’. This suggestion was made on the basis that students have different family conventions and many do not live with their family. Therefore, ‘community’ may be a more appropriate term as it could relate to any person a student resides with. This suggestion is important as it reflects the diverse living situations and social structures of students. These results are consistent with previous authors who indicated that many students may not live with their traditional family or may define their support system differently [48,49]. Thus, by using the term ‘community,’ the guidelines become more inclusive and relevant to students, acknowledging that support may stem from various sources, such as roommates, siblings, or other close connections [50]. This terminology change ensures that the guidelines are more applicable and supportive in promoting a sense of connectedness, camaraderie, and well-being [51].

### 4.1. Strengths and Limitations

This research demonstrates several strengths. To the author’s knowledge, this is the first study to systematically employ a consensus workshop approach in SA to develop context-specific PA guidelines tailored specifically for undergraduate university students. By focusing exclusively on a single university in the Western Cape Province, the study provides a detailed examination of the unique PA and psychosocial dynamics, ensuring contextual relevance. The use of a mixed-methodological design, alongside a consensus workshop approach, offers a comprehensive method for developing tailored PA guidelines. Stakeholder engagement was maximized by leveraging technology, such as breakout rooms within Google Meets, to facilitate asynchronous discussions, which allowed participants to contribute to the discussion, thus reducing the need for numerous workshops. Furthermore, this study stands out as one of the pioneering efforts in SA to employ the consensus workshop method specifically for crafting PA guidelines, highlighting its potential influence on future public health interventions. This study’s innovative use of the consensus workshop method provides an evidence-based framework with significant potential to influence public health interventions and policies. By incorporating stakeholder input, this approach ensures that context-specific guidelines are tailored to the needs of university students, offering a model for inclusive and practical intervention design. Its adaptability and emphasis on collaboration make it a valuable tool for developing sustainable interventions that address sedentary behavior and promote PA participation among students.

Although this research adds a novel contribution to academia, some limitations must be acknowledged. One limitation of the study was the difficulty in arranging a time and date for the consensus workshop that accommodated both undergraduate university students and academic doctors and professors. Many stakeholders, particularly undergraduate students, have lectures and academic commitments that may conflict with the workshop schedule. Similarly, professors and academic doctors have professional obligations that make finding a mutually convenient time challenging. These scheduling challenges may result in lower participation rates or the exclusion of some key stakeholders. Therefore, future studies should consider conducting consensus workshops during semester breaks or term holidays, when lecture classes have been concluded. Thus, stakeholders would have more time to participate in the workshop. It is also acknowledged that the majority of student participants were from sports-related programs, which may have shaped their perspectives on PA terminology. This concentration in sport-focused disciplines could limit the generalizability of the findings, as students from a broader range of academic fields might have provided more varied insights. Therefore, future research should aim to include a more diverse sample of students to offer a more comprehensive understanding of the terminology used to develop PA guidelines. Additionally, a limitation of this study is the potential for challenges in scaling the workshop model to larger more diverse populations, as well as the implications of conducting the workshops online. The online format may have influenced participant engagement and interaction, which could differ from in-person sessions. Another limitation of the consensus workshop process is the potential variability in participants’ prior knowledge and perspectives, which may have influenced the guidelines’ development. As the workshop involved a select group, the findings may not fully reflect the diversity of the views among all undergraduate university students, potentially limiting the guidelines’ broader applicability and acceptance. Future research could address this by including a more diverse participant group. Lastly, the study was conducted at one university in the Western Province region of SA. Therefore, the findings may not be generalizable to other regions. To mitigate this limitation, future research should involve additional universities and postgraduate students as part of the inclusion criteria to obtain a more representative and diverse range of perspectives.

### 4.2. Recommendations

Based on the results of this study, the following recommendations are provided to improve undergraduate students’ PA engagement. Future studies could expand on the current research by increasing the sample size to include participants from multiple universities across SA. This would allow for the collection of diverse perspectives, as different universities may have unique contexts, providing a more comprehensive understanding of physical inactivity. For practitioners, it is recommended that experts in the fields of PA, sports science, and public health build on the insights derived from these consensus workshops and incorporate these guidelines into their practices, focusing on enhancing PA among undergraduate university students. In terms of SA universities, these guidelines could be incorporated into existing university programs, such as student orientation, PA health and wellness campaigns, and academic courses. Universities could offer flexible low-barrier PA options that align with students’ schedules and preferences, making participation more accessible and appealing. For example, integrating PA sessions during breaks, between classes, or offering evening and weekend activities could increase student engagement. Moreover, universities should collaborate with departments such as sports science, public health, and student support services to ensure the guidelines are embedded within extracurricular activities, health education workshops, and peer-led initiatives. Student health clubs and recreational sports leagues may also utilize the guidelines to design targeted programs that encourage sustained engagement in PA. Furthermore, the Department of Higher Education (DHET) in SA could utilize the guidelines generated herein as a framework to design and develop student-tailored PA initiatives. By implementing these guidelines, universities could proactively enhance PA participation, ultimately contributing to achieving Sustainable Development Goal 3, which focuses on ensuring healthy lives and promoting well-being among the student population in the context of this study.

### 4.3. Future Research

Future studies may build on the current study by conducting longitudinal research to assess the long-term effectiveness of the PA guidelines developed through the consensus workshop. By monitoring and evaluating undergraduate university students’ PA over time, researchers could track the sustainability of these interventions. Additionally, researchers could compare the outcomes of virtual versus in-person consensus workshops. This may provide valuable insights into how the mode of participation influences the depth of the stakeholder discussions and engagement as well as the overall quality of the guidelines that are produced. Lastly, to enhance stakeholder engagement, alternative approaches could complement the consensus workshop method. For instance, participatory action research (PAR) could involve stakeholders more directly in the research process, allowing them to co-create and refine the guidelines based on their lived experiences. Additionally, Delphi techniques, with iterative rounds of feedback from stakeholders, could further refine the outcomes by gathering a broader range of perspectives to ensure that the voices of diverse stakeholders are heard. These approaches would not only enrich stakeholder engagement but also lead to more robust and contextually relevant guidelines. These approaches would not only enhance the effectiveness of the PA guidelines but also offer critical insights into optimizing the consensus workshop process for developing impactful context-specific PA initiatives.

## 5. Conclusions

Employing a consensus workshop provided multifaceted information to support the co-creation of context-specific guidelines to enhance the physical activities of undergraduate university students. This collaborative method provided stakeholders (i.e., undergraduate university students and exercise/public health-related experts) with the opportunity to understand the research interventions and findings and to discuss and or critique the results based on their experiences [52]. This enabled stakeholders to be involved in the decision-making process. Moreover, by engaging with stakeholders in this co-creative and collaborative process, the guidelines tailored to students’ preferences would be context-specific and pertinent to today’s undergraduate student population. The results of this study may be useful to students and policymakers in addressing the concern of physical inactivity.

## Figures and Tables

**Table 1:** Findings from the mixed-methodological approach, utilizing the sequential explanatory research design, were captured and reorganized into guidelines within the adapted version of the Social Ecological Model framework.

Topics from the Main Findings	Central Theme	Guidelines	
**Quantitative** **PA** 29% of undergraduate students were physically inactive. 31.1% were minimally active. 39.9% were in the health-enhancing category. Evening sessions were preferred. Endurance activities were favoured. **PA Facilitators:** Social support. Social media. Recognition from others. **PA Barriers:** Lack of knowledge. Time constraints. Financial limitations. **Psychosocial factors** PA was positively related to stress (r = 0.11, *p* < 0.05). PA was positively related to anxiety (r = 0.10, *p* < 0.05). Motivational factors were positively related to psychological conditions and others’ expectations (r = 0.10, *p* < 0.05). Motivational factors were positively related to depression and others’ expectations (r = 0.11, *p* < 0.05). 23.2% of students experienced extremely severe depression. 40.6% of students experienced extremely severe anxiety **Qualitative** **Motivation** Improving body shape. Finding happiness. Achieving personal best. **Social Support** Peers and friends provided motivation, encouragement, and accountability. Family members offered encouragement, constructive criticism and financial support. Health experts were valued for their guidance and health-related knowledge. Peer pressure was identified as a negative influence on PA participation. **Physical Environment** Students preferred engaging in PA both on and off campus. Accessibility, affordability and safety of the environments influenced preferences. *Social Media* Instagram, Facebook and YouTube were used for communication, education, and staying connected. Some students spent up to 10 h daily on these platforms. Efficient communication. Access to information.	Physical Activity	Guideline 1: Start with small beginnings Guideline 2: Increase activity levels gradually Guideline 3: Sustain and diversify activities Guideline 4: Monitor yourself with the talk test Guideline 5: Schedule PA strategically Guideline 6: Choose preferred activities Guideline 7: Monitor your progress Guideline 8: Pursue health education Guideline 9: Track time usage Guideline 10: Seek affordable, convenient engagement	
	Psychosocial factors	SEM level	Guidelines
	Mental Health	Individual	Guideline 1: Balance intrinsic and extrinsic motivation Guideline 2: Utilise PA as a buffer against depression Guideline 3: Include anxiety-reducing activities Guideline 4: Introduce stress-relieving activities
		Social	Guideline 5: Build a support network
		Physical Environment	Guideline 6: Utilise serene environments
		Public Engagement	Guideline 7: Be mindfulGuideline 8: Establish healthy screen time
	Motivation	Individual	Guideline 1: Master fitness goals and competence (intrinsic motivation) Guideline 2: Maintaining health Guideline 3: Sustain motivation with enjoyable activities
		Social	Guideline 4: Focus on personal well-being Guideline 5: Meet your own standards
		Physical Environment	Guideline 6: Active study spaces
		Public Engagement	Guideline 7: Prioritise health over appearance Guideline 8: Avoid imitating others Guideline 9: Mindful social media engagement Guideline 10: Engage in Social Media challenges for motivation
	Social Support	Individual	Guideline 1: Identify positive support networks
		Social	Guideline 2: Foster family fitness Guideline 3: Cultivate a supportive fitness community through positive peer associations
		Physical Environment	Guideline 4: Campus-based social support Guideline 5: Utilise campus resources
		Public Engagement	Guideline 6: Consult health experts Guideline 7: Use social media to your advantage

Note: PA = Physical Activity, SEM = Social Ecological Model.

**Table 3:** Round 1 of recommended guidelines with comments from the stakeholders.

Central Theme	Guidelines		Stakeholder(s) Comments
**Physical Activity**	Guideline 1: Start with small beginnings		“Agree with the guideline. Clearly note to the participants that all the modalities used are purely exercised based and have no link to any form of religion or spiritual background.
	Guideline 2: Increase activity levels gradually		“Agree with the guideline. The emphasis should also be on GRADUALLY and we would likely change the name to foreground that”.
	Guideline 3: Sustain and diversify activities		“Agree with the guideline”.
	Guideline 4: Monitor yourself with the talk test		“Agree with the guideline”.
	Guideline 5: Schedule PA strategically		“Agree with the guideline”.
	Guideline 6: Choose preferred activities		“Agree with guideline but seems it should be moved further up”.
	Guideline 7: Monitor your progress		“Agree with the guideline”.
	Guideline 8: Pursue health education		“Agree with the guideline”.
	Guideline 9: Track time usage		“Agree with the guideline”.
	Guideline 10: Seek affordable, convenient engagement		“Link to monitoring”
**Psychosocial factors**	SEM level	Guidelines	
**Mental Health**	Individual	Guideline 1: Balance intrinsic and extrinsic motivation	“Agree with the guideline. Exercise is tough. One needs the motivation to engage in PA”.
		Guideline 2: Utilise PA as a buffer against depression	“Change to: Utilise PA as a coping mechanism for depression”.
		Guideline 3: Include anxiety-reducing activities	“Agree with the guideline. A layman will struggle with differentiating depression, stress and anxiety. A short contextual description will help”.
		Guideline 4: Introduce stress-relieving activities	“Why “introduce” rather than “include”?”
	Social	Guideline 5: Build a support network	“Wouldn’t “Be a part of or join a PA support group network” be more comprehensive?”
	Physical Environment	Guideline 6: Utilise serene environments	“Agree with the guideline”.
	Public Engagement	Guideline 7: Be mindful	“Agree with the guideline”.
		Guideline 8: Establish healthy screen time	“Agree with the guideline”.
**Motivation**	Individual	Guideline 1: Master fitness goals and competence (intrinsic motivation)	“Reconsider rewording the guideline to ‘Master motivational goals’”.
		Guideline 2: Maintaining health	“Consider: Motivation to maintain health”.
		Guideline 3: Sustain motivation with enjoyable activities	“Agree with the guideline”.
	Social	Guideline 4: Focus on personal well-being	“Agree with the guideline”.
		Guideline 5: Meet your own standards	“Agree with the guideline”.
	Physical Environment	Guideline 6: Active study spaces	“Agree with the guideline”.
	Public Engagement	Guideline 7: Prioritise health over appearance	“Consider: prioritise health over physical appearance”.
		Guideline 8: Avoid imitating others	“Consider: changing the word imitating to copying”.
		Guideline 9: Mindful of social media engagement	“Consider: Be mindful of social media influences”
		Guideline 10: Engage in social media challenges for motivation	“Consider: Engage in social media fitness challenges for motivation”
**Social Support**	Individual	Guideline 1: Identify positive support networks	“Agree with the guideline”.
	Social	Guideline 2: Foster family fitness	“Agree with the guideline”.
		Guideline 3: Cultivate a supportive fitness community through positive peer associations	“Agree with the guideline. Be inclusive towards friends in different stages of their wellness”.
	Physical Environment	Guideline 4: Campus-based social support	“Agree with the guideline”.
		Guideline 5: Utilise campus resources	“Agree with the guideline”.
	Public Engagement	Guideline 6: Consult health experts	“Agree with the guideline”.
		Guideline 7: Use social media to your advantage	“Agree with the guideline”.

Note: PA = Physical Activity, SEM = Social Ecological Model.

**Table 4:** Round 2 of recommended guidelines with comments from the stakeholders.

Central Theme	Guidelines		Stakeholder(s) Comments	Consensus (%)
**Physical Activity**	Guideline 1: Start with small beginnings		“Agree with the guideline. Integrate for each activity (or as an overall part of the guide) that persons with mobility impairments can explore alternative exercises that are safe—in this way your guideline shows disability inclusion”.	100
	Guideline 2: Gradually increase activity levels		“Agree with the guideline”.	100
	Guideline 3: Sustain and diversify activities		“Agree with the guideline”.	100
	Guideline 4: Choose preferred activities		“Agree with the guideline”.	100
	Guideline 5: Monitor yourself		“Agree with the guideline”.	100
	Guideline 6: Track time usage between PA and academic commitments		“Agree with the guideline”.	100
	Guideline 7: Schedule PA strategically		“Agree with the guideline”.	100
	Guideline 8: Pursue health education		“Agree with the guideline. Propose to move this Guideline to #1 for PA, so that people understand this as a first step”.	100
	Guideline 9: Affordable, convenient engagement		“Agree with the guideline”.	100
**Psychosocial factors**	SEM level	Guidelines		
**Mental Health**	Individual	Guideline 1: Balance intrinsic and extrinsic motivation	“Agree with the guideline. Mental health fluctuates daily, therefore, we think PA as a way to take care of your mental health should be considered”.	100
		Guideline 2: Utilise PA as a coping mechanism to deter depression	“Rather say, ‘Utilise PA as a coping mechanism for depression’.	94.1
		Guideline 3: Include anxiety-reducing activities	“Agree with the guideline”.	100
		Guideline 4: Include stress-relieving activities	“Agree with the guideline”.	100
	Social	Guideline 5: Join a PA support network group	“Agree with the guideline. Conversations need to be held around how beneficial it is for everyone’s mental health”.	100
	Physical Environment	Guideline 6: Utilise serene environments	“Agree with the guideline”.	100
	Public Engagement	Guideline 7: Mindful screen management for productivity and well-being	“Agree with the guideline”.	100
**Motivation**	Individual	Guideline 1: Master motivational goals and build your competence (intrinsic motivation)	“Change to ‘Set (or some other term) motivational goals and activities’. ‘Master’ is too strong”.	94.1
		Guideline 2: Motivation to maintain health	“Agree with the guideline”.	100
		Guideline 3: Incorporate enjoyable activities	“Agree with the guideline”.	100
	Social	Guideline 4: Focus on a healthy lifestyle	“Agree with the guideline”.	100
	Physical Environment	Guideline 5: Use active study spaces	“Change to ‘Identify and utilise active learning spaces”. ‘Learning’ is a more appropriate term”.	88.2
	Public Engagement	Guideline 6: Prioritise health over external appearance	“Change to ‘Make your health as important as your appearance’ Keep it simple”.	88.2
		Guideline 7: Be yourself	“Agree with the guideline”.	100
		Guideline 8: Foster a healthy mindset when engaging in social media	“Agree with the guideline”.	100
		Guideline 9: Engaging in positive social media fitness challenges can be motivational	“Agree with the guideline”.	100
**Social Support**	Individual	Guideline 1: Surround yourself with PA support networks	“Agree with the guideline”.	100
	Social	Guideline 2: Foster family fitness	“Rename to ‘Promote community wellness’. Students might not be living with their families or just have very different family conventions. By perhaps using the word community—this includes who you are currently living with”.	88.2
		Guideline 3: Cultivate a supportive fitness community through positive peer associations	“Agree with the guideline”.	100
	Physical Environment	Guideline 4: Identify a PA that will foster social comfort	“Agree with the guideline. It is wonderful having a community that will support you during PA and socially”.	100
		Guideline 5: Utilise campus resources	“Rename to ‘Utilise campus recreational or fitness resources’. This emphasises the PA aspect”.	88.2
	Public Engagement	Guideline 6: Consult health experts for your fitness journey	“Agree with the guideline”.	100
		Guideline 7: Use social media for fitness motivation	“Agree with the guideline”.	100

Note: PA = Physical Activity, SEM = Social Ecological Model.

## Data Availability

The data presented in this study are available on request from the corresponding author. The data are not publicly available due to ethical restrictions.
